# Supplementary material for: State-specific projection of COVID-19 infection in the United States and evaluation of three major control measures
Source: Sci Rep. 2020 Dec 30;10:22429. doi: 10.1038/s41598-020-80044-3 (PMC7773742; doi:10.1038/s41598-020-80044-3)
Supplement: Supplementary file 1 — Supplementary material 1 [file 41598_2020_80044_MOESM1_ESM.pdf]

Supporting Material for "State-specific Projection of COVID-19 Infection in the United States and Evaluation of Three Major Control Measures"

Shi Chen[1], Qin Li[1,*], Song Gao[2,*], Yuhao Kang[2], and Xun Shi[3]

[1]Department of Mathematics, University of Wisconsin-Madison, Madison, WI 53706, USA
[2]GeoDS Lab, Department of Geography, University of Wisconsin-Madison, Madison, WI 53706, USA
[3]Department of Geography, Dartmouth College, Hanover, NH 03755, USA
[*]Corresponding Authors: qinli@math.wisc.edu; song.gao@wisc.edu.

April 2, 2020

1 Mathematical model and configuration

The mathematical model used in this article is derived from the susceptible-exposed-infectious-removed (SEIR) [7, 6, 2, 8] augmented to incorporate human movement, and separate the infected compartment into reported cases and unreported cases. The model is illustrated in the flowchat below:

In the model, for each state, the population is divided into six compartments: S_i(susceptible), E_i(latent), I_i(reported infections), U_i(unreported infections) and R_i(removed: either recovered or deceased individuals). The subindex i stands for the index for the state. Among the six compartments, S, E and U are "free" people and can move from state to state, while I, and R are monitored and isolated.

The model is composed of 51 coupled ordinary differential equation (ODE) systems. For each

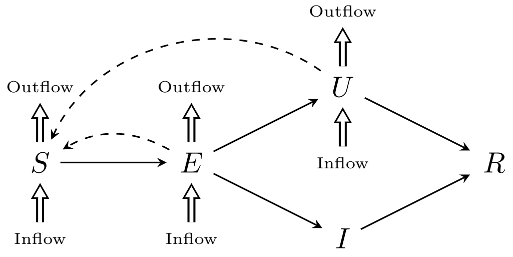

Figure S1: Illustration of the travel flow-network augmented susceptible-exposed-infectious-removed model.

state i, the model writes as:

$$
\begin{cases}
\dfrac{\mathrm{d}S_i}{\mathrm{d}t} = -\dfrac{b_i S_i (U_i + \gamma E_i)}{P_i} + \sum_{j\neq i} \alpha_t n_{ij} \dfrac{S_j}{P_j} - \sum_{j\neq i} \alpha_t n_{ji} \dfrac{S_i}{P_i} \\
\dfrac{\mathrm{d}E_i}{\mathrm{d}t} = \dfrac{b_i S_i (U_i + \gamma E_i)}{P_i} - \dfrac{E_i}{D_e} + \sum_{j\neq i} \alpha_t n_{ij} \dfrac{E_j}{P_j} - \sum_{j\neq i} \alpha_t n_{ji} \dfrac{E_i}{P_i} \\
\dfrac{\mathrm{d}I_i}{\mathrm{d}t} = r_i \dfrac{E_i}{D_e} - c_I \dfrac{I_i}{D_c} - (1-c_I)\dfrac{I_i}{D_l} \\
\dfrac{\mathrm{d}U_i}{\mathrm{d}t} = (1-r_i)\dfrac{E_i}{D_e} - c_U \dfrac{U_i}{D_c} - (1-c_U)\dfrac{U_i}{D_l} + \sum_{j\neq i} \alpha_t n_{ij} \dfrac{U_j}{P_j} - \sum_{j\neq i} \alpha_t n_{ji} \dfrac{U_i}{P_i} \\
\dfrac{\mathrm{d}R_i}{\mathrm{d}t} = c_I \dfrac{I_i}{D_c} + (1-c_I)\dfrac{I_i}{D_l} + c_U \dfrac{U_i}{D_c} + (1-c_U)\dfrac{U_i}{D_l}
\end{cases}
. \quad (1)
$$

The ODE system is equipped with the following initial data ($t = 0$ standing for March 1, 2020):

$$S_i(0) = N_i - E_{i0} - U_{i0} - I_{i0}\,, \quad E_i(0) = E_{i0}\,, \quad I_i(0) = I_{i0}\,, \quad U_i(0) = U_{i0}\,, \quad R_i(0) = 0. \quad (2)$$

In the equation, the unit for t is one day. $N_i(t)$ is the total population of state i at time t, and $P_i = S_i + E_i + U_i$ is the free population. n_{ij} is the number of inflow from state j to state i. b_i and r_i are the transmission rate and reporting rate of state i. c_I (c_U, resp.) is the proportion of positive cases that show critical condition for I (unreported cases U, resp.). D_e is the latent period. D_c and D_l are the infectious periods of critical cases and mild cases. α_t is a parameter to tune the traffic flow.

We emphasize two main differences in modeling compared with literature. In [10], the authors study the inter-city traffic and its impact on the spreading of COVID-19 in China. The situation in China and that in the US are very different. In China, the epicenter is clear: the city of Wuhan, Hubei province, and the outbreak starts mid-January, 2020. The COVID-19 outbreak in the US, however, is multi-sourced. The consequence is that in the model in [10], the initial condition for cities excepts Wuhan is clear: the latent, the reported and the unreported cases are all zero. In this model, however, the initial conditions E_{i0} are unclear for all states; Another big difference is, according to clinical findings, the latent cases also have the potential of transmitting the virus, and thus we add the interaction of E_i with S_i into the increment of E_i [9, 10, 1].

The unknown parameters and state variables in the equation set are

* b_i: the transmission rate with non-informative prior range $[1, 1.5]$;
* r_i: the report rate with non-informative prior range $[0.1, 0.3]$;
* E_{i0}: the data for the latent population with non-informative prior range $[0, 500]$.
* U_{i0}: the initial data for the unreported population with non-informative prior range $[0, 200]$.
* S_{i0}: the initial data for the susceptible population defined by $N_i - E_{i0} - I_{i0} - U_{i0}$.

Other parameters are:

γ: the transmission ratio between unreported and latent. In the simulation we set it to be 0.5;

D_c: the average duration of infection for critical cases. We assume $D_c = 2.3$ days [5].

D_e: the average latent period. According to [13], $D_e = 5.2$ days.

D_l: the average duration of infection for mild cases. We assume $D_l = 6$ days.

α_t: the ratio of interstate travel volume compared to that of 2019 during the same period. The travel flow information n_{ij} was extracted from the SafeGraph mobility data, and we set $\alpha_t = 0.5$ to represent the travel reduction situation observed in the year of 2020.

c_I: proportion of critical cases among all reported cases. We assume $c_I = 0.1$.

c_U: proportion of critical cases among all unreported cases. We assume $c_U = 0.2$.

There is an essential assumption made in the model: the homogeneity in the population. It means that the traffic flow is a good representation of the total population without considering their demographic and socioeconomic characteristics. The susceptible, exposed, and unreported move in and out of states at the same rate. This explains the $\frac{S_i}{P_i}$, $\frac{E_i}{P_i}$ and $\frac{U_i}{P_i}$ terms in the $S_i/E_i/U_i$ equation.

The effective reproductive number R_e could be computed as

$$R_e = \frac{b}{E+U}\left[\gamma D_e E + \frac{D_c D_l U}{c_U D_l + (1-c_U)D_c}\right]. \quad (3)$$

R_e depends on time due to the time dependence of E and U.

2 Data acquisition

2.1 COVID-19 Observation Data

The only available data in the $(S-E-I-U-R)$ system, for every state, is I, the reported confirmed cases. We fetch the data from a community-developed, open source project: Novel Coronavirus (COVID-19) Cases, developed by the Center For Systems Science and Engineering at the Johns Hopkins University [3] [1].

2.2 Population and Human Mobility Data

We downloaded the total population data by state in 2019 from the US Census Bureau. In addition, we collected over 3.6 million points of interest (POIs) with travel patterns in the United States from the SafeGraph business venue database[2]. The SafeGraph's data sampling correlated highly with the United States Census populations[3]. These mobile location data consist of "pings" identifying the coordinates of a smartphone at a moment in time. To enhance privacy, SafeGraph excludes census block group (CBG) information if fewer than five devices visited a place in a month from a given CBG. For each POI, the records of aggregated visitor patterns illustrate the number of unique visitors and the number of total visits to each venue during the specified time window (i.e., March 1st to March 31st 2019 in our dataset), which could reflect the attractiveness of each venue and the national spatial interaction patterns during the last March travel. According to the ODE modeling needs, we further aggregated the travel patterns to the state-to-state spatial scale as shown in Figure S2. In the model, we set the parameter $\alpha_t = 0.5$ to represent the travel reduction situation observed in the year of 2020 [14].

[1] https://github.com/CSSEGISandData/COVID-19/blob/master/csse_covid_19_data/csse_covid_19_time_series/time_series_covid19_confirmed_global.csv

[2] www.safegraph.com

[3] www.safegraph.com/blog/what-about-bias-in-the-safegraph-dataset

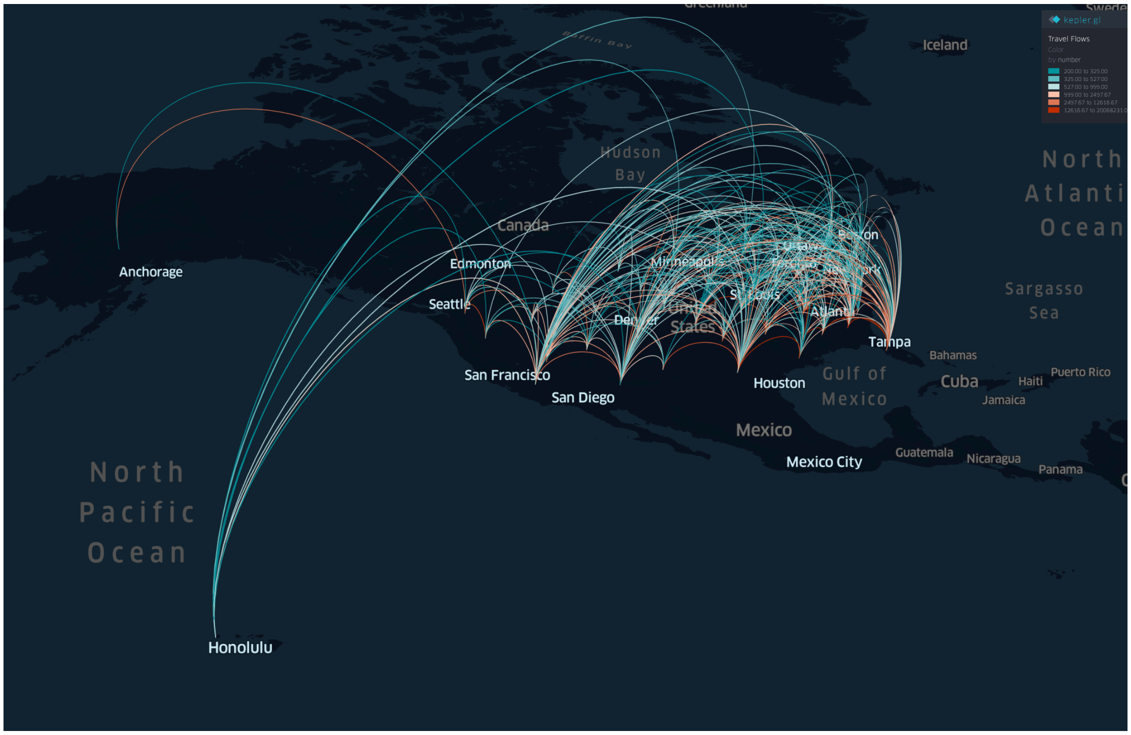

Figure S2: The map of US state-to-state travel patterns aggregated in March 2019 (Data source: SafeGraph; Visualization: kepler.gl)

.

3 Parameter fitting methodology

Each state has its own $S-E-I-U-R$ data. We assume all the states have their own transmission rate b, and the reporting rate r. In this section we discuss the method we apply to recover these parameters.

To identify the parameters is a typical data assimilation problem: one has the knowledge from an underlying ODE model, and the access to evolution data. The goal is to build a probability density function that reveals the possible value and the probability of the state variable. Two main ingredients in DA are ODE simulation, and the Bayesian analysis. The ODE system serves as the prior information, and the Bayesian formula blends such dynamical system with the newly fetched data to generate a posterior distribution of the state variables and the parameters. The general flow chart of data assimilation is found in Figure S3.

In the flowchart, $P_{n|n-1}$ is the probability density of u upon the evolution step, and $P_{n|n}$ is the probability density obtained through the analysis step.

In our case, u is the augmented state variable that includes both the unknown parameters (b, r) and the state variable (I, E, S, U, R) for every state:

$$u = (b_i, r_i, I_i, E_i, S_i, U_i, R_i)^\top \in \mathbb{R}^{7k}\,,$$

where k is the number of states/regions used in the fitting. We transpose it to make it into a column vector. We are aiming at building a distribution density function of u over the $\mathbb{R}^{7k}$ space. $\mathcal{G}_{m,n}(u)$ is the solution to the ODE (1) at time t_n with u serving as the parameter in the equation

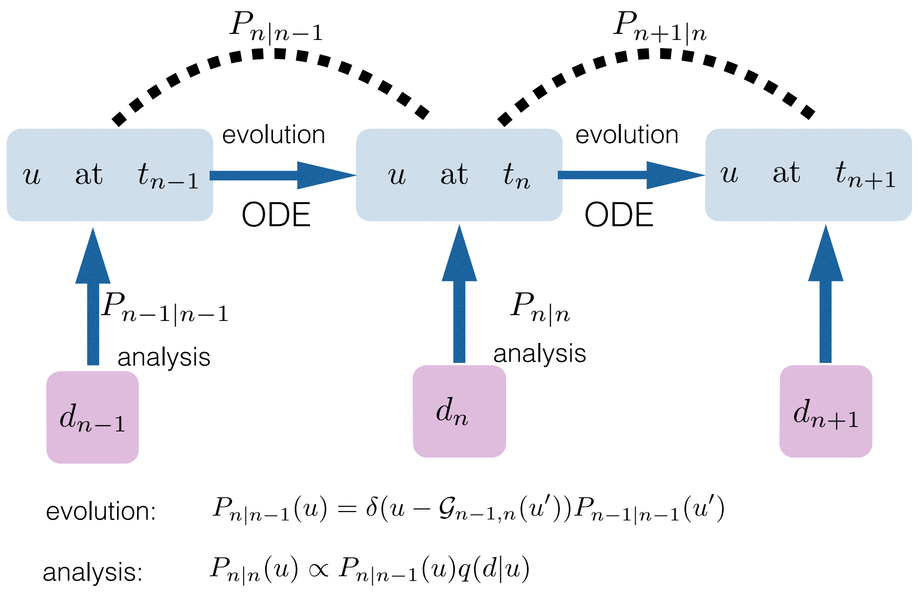

Figure S3: Flowchart of data assimilation: u is the state variable, $\mathcal{G}_{n-1,n}(u)$ is the forward map by running the ODE from time step t_{n-1} to t_n. q is the likelihood function that measures the probability of the error term $d - \mathcal{M}u$ where $\mathcal{M}$ is the measuring operator.

at time t_m. The measuring operator is $\mathcal{M}(u) = [I_i]_{i=1}^k$, and thus we denote

$$\mathcal{M}(u) = M \cdot u \,, \quad \text{with} \quad M = \otimes^k [0,0,1,0,0,0]] \,,$$

meaning M is a $7k$ length vector that has k nontrivial entries that pick up all I_i information.

We further assume the collected data has a Gaussian perturbation from the true measuring operator:

$$d_n = \mathcal{M}(u_n) + \eta \,, \quad \text{with} \quad \eta \sim \mathcal{N}(0, \sigma^2) \,,$$

and thus naturally the likelihood function is:

$$q(d|u) = \frac{1}{2\pi\sigma} e^{-\frac{|d-\mathcal{M}(u)|^2}{2\sigma^2}} \,.$$

Then together with the two formula for the evolution step and the analysis step:

– Evolution: $P_{n|n-1}(u) = \delta(u - \mathcal{G}_{n-1,n}(u'))P_{n-1|n-1}(u')$

– Analysis: $P_{n|n}(u) \propto P_{n|n-1}(u)q(d|u)$

one can iteratively update $P_{n|n}(u)$, giving $P_{n-1|n-1}$, the probability density of u at time t_{n-1}. In the equation $\propto$ is the proportional sign: one needs to normalize $P_{n|n}$ to make it a probability density function so that $\int P_{n|n}(u)\mathrm{d}u = 1$.

There are many choices of data assimilation methods. We choose to utilize Ensemble Kalman Filter that is steered towards analyzing systems having high dimensional state variables. It is a technique, derived from the classical Kalman Filter for the application in atmospheric science, with the analytical covariance matrix replaced by the ensemble version, eliminating the computation of the the Kalman gain matrix and the Riccati Equation that are typically expensive in high dimensional space. It is proved to the effective in the Gaussian case for linear forward model

and the measuring operator [4, 11, 12]. The idea is to sample a fixed number of particles on the state variable space according to the initial distribution, and move these particles around at every discrete time step with certain dynamics, to represent the newly adjusted distribution. Denote the number of particles by N, and the j-th particle after the evolution step at time t_n by $u_n^{j,\mathrm{E}}$, and the j-th particle after the analysis step at t_n by $u_n^{j,\mathrm{A}}$, we now summarize the algorithm:

– Evolution:
$$u_n^{j,\mathrm{E}} = FE(\mathcal{G}_{n-1,n}(u_{n-1}^{j,\mathrm{A}}))\,.$$
where FE is the Forward-Euler discretization applied on ODE (1).

– Analysis:
$$u_n^{j,\mathrm{A}} = u_n^{j,\mathrm{E}} + \mathrm{Cov}_n^{\mathrm{up}}\left(\mathrm{Cov}_n^{\mathrm{pp}} + \sigma^2\right)^{-1}\left(d_n + \xi_n^j - \mathcal{M}u^{j,\mathrm{E}}\right),$$
where ξ_n^j is a k-length vector with each entry being random variable i.i.d. drawn from $\mathcal{N}(0,\sigma^2)$, d_n is the k-length vector collecting the reported infected data on k counties. To compute the covariance matrices, we set:
$$\bar{u}_n^{\mathrm{E}} = \frac{1}{N}\sum_{j=1}^{N} u_n^{j,\mathrm{E}}\,,\quad \mathrm{Cov}_n^{\mathrm{uu}} = \frac{1}{N}\sum_{j=1}^{N}(u_n^{j,\mathrm{E}} - \bar{u}_n^{\mathrm{E}})(u_n^{j,\mathrm{E}} - \bar{u}_n^{\mathrm{E}})^\top\,.$$
Then naturally
$$\mathrm{Cov}_n^{\mathrm{up}} = \mathrm{Cov}_n^{\mathrm{uu}}\cdot M^\top\,,\quad \mathrm{Cov}_n^{\mathrm{up}} = M\cdot\mathrm{Cov}_n^{\mathrm{uu}}\cdot M^\top\,.$$
are matrices of size $7k \times k$ and $k \times k$ respectively.

4 Results

The results are divided into two categories: 1. parameter fitting; 2. COVID-19 infection prediction. The computation is done on the state level. Data from 50 states and D.C. (thus $k = 51$) in the United States are used. The model and data assimilation analyses were ran from March 1 to March 20, 2020, and we predict the future infectious cases in different states from March 21 to April 29, 2020 with different parameter setting scenarios.

4.1 State results

For parameter fitting, we utilize the method discussed in Section 3. Total 2000 samples with non-informative prior are adopted to determine $7 \times 51 = 357$ state variables. The standard deviation of noise is set to be $\sigma = 10$. In Figure S4-S5, we plot the susceptible, exposed, unreported infections and removed, in time, for the top 10 states with most total confirmed cases as of March 20, respectively. In Figure S6-S7, we plot the reported infections in time, for these 10 states. For most states, the number of reported case grows essentially exponentially fast. Figure S8-S11 show the inferred transmission rate b and reported rate r. Figure S12-S13 show the time series of effective reproductive number R_e for different states. The signal in the data is rather weak, and for some states, the number of E and U cannot be inferred at the early stage of the breakout, leading to $R_e = 0$ for a small period of time for some states.

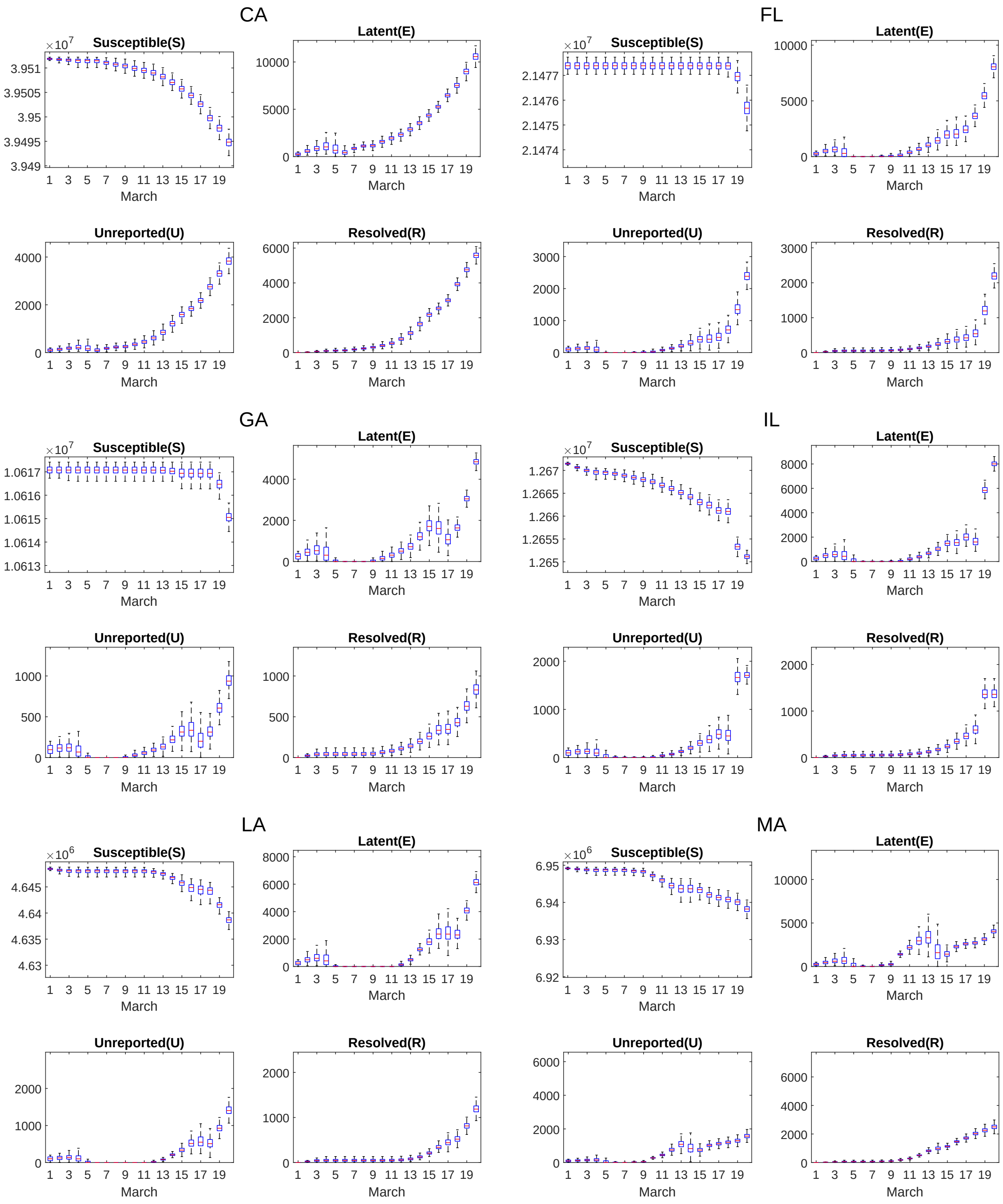

Figure S4: Simulation of $S - E - U - R$ for different states. The box and whiskers show the median, interquartile range, and 95% credible intervals.

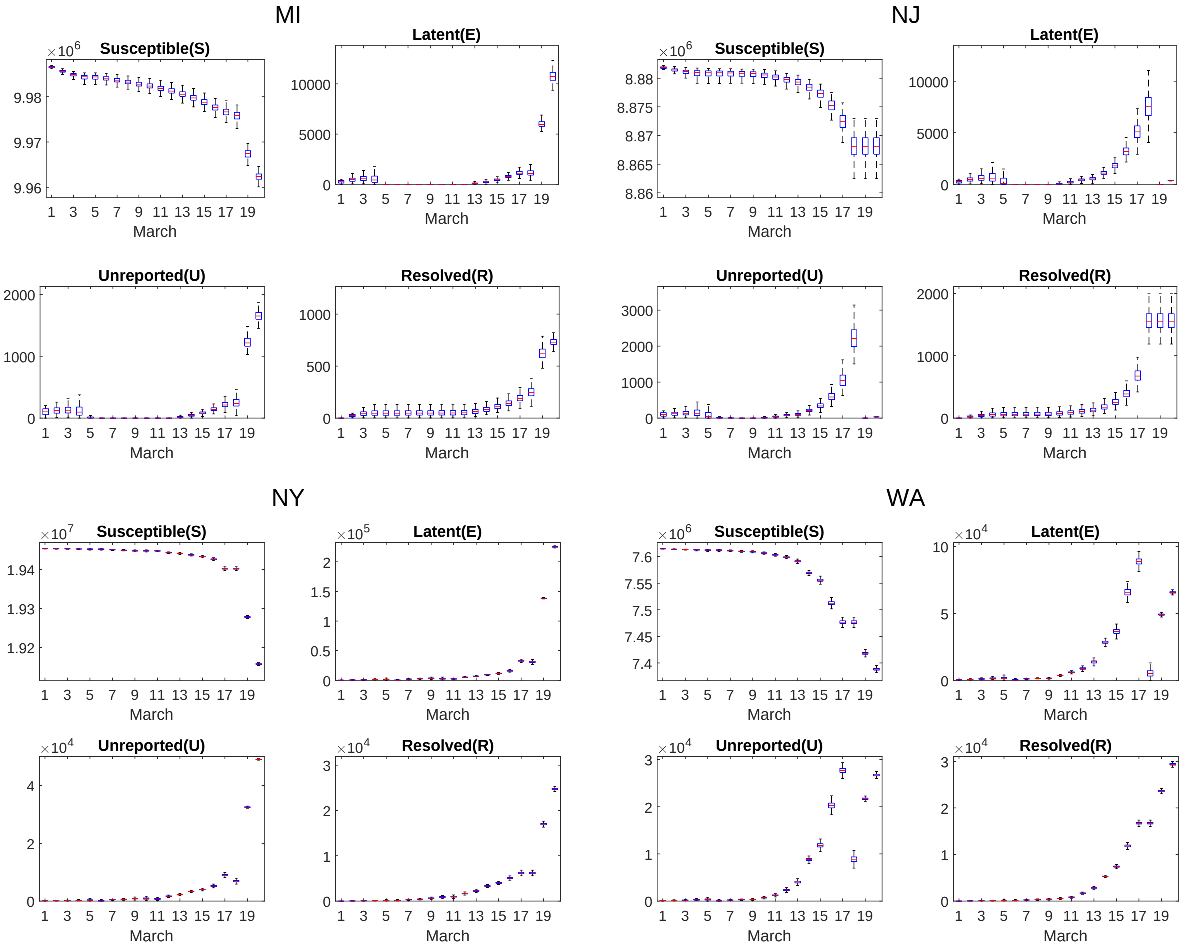

Figure S5: Simulation of $S - E - U - R$ for different states. (continued)

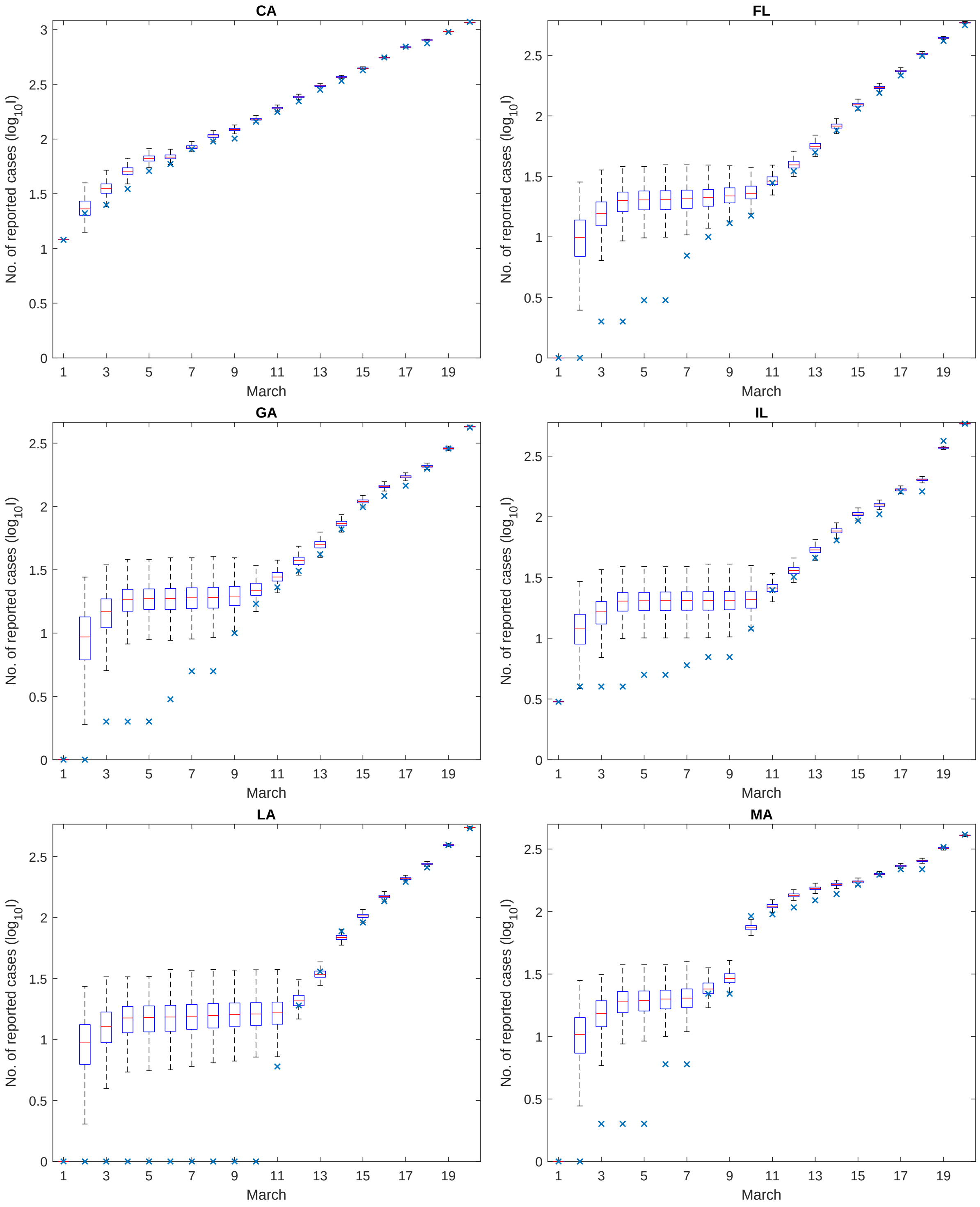

Figure S6: Simulation of confirmed cases I (boxes) and the true confirmed cases I_{true} (blue x's) for different states. The box and whiskers show the median, interquartile range, and 95% credible intervals.

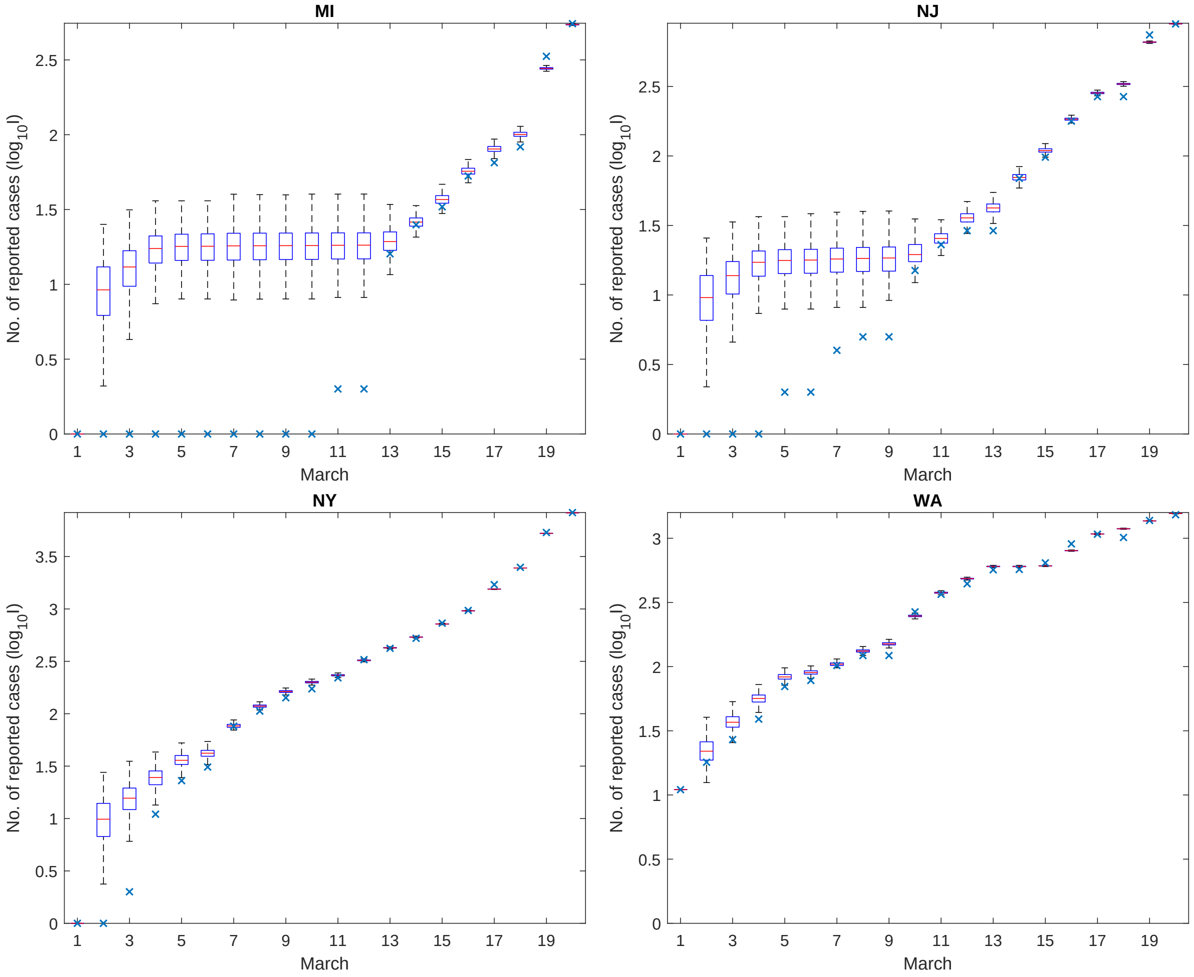

Figure S7: Simulation of confirmed cases I (boxes) and the true confirmed cases I_{true} (blue x's) for different states. (continued)

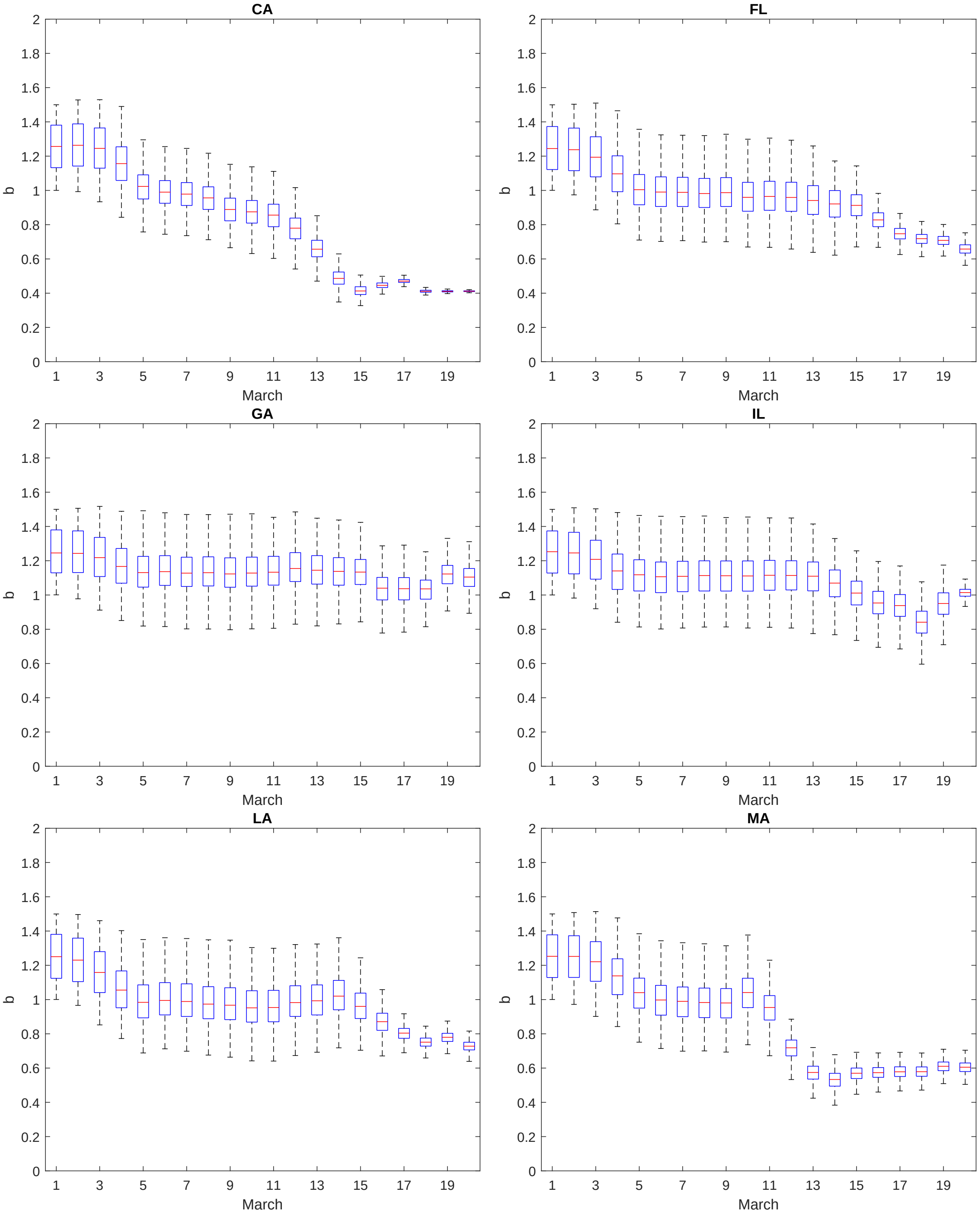

Figure S8: Inferred transmission rate b for different states. The box and whiskers show the median, interquartile range, and 95% credible intervals.

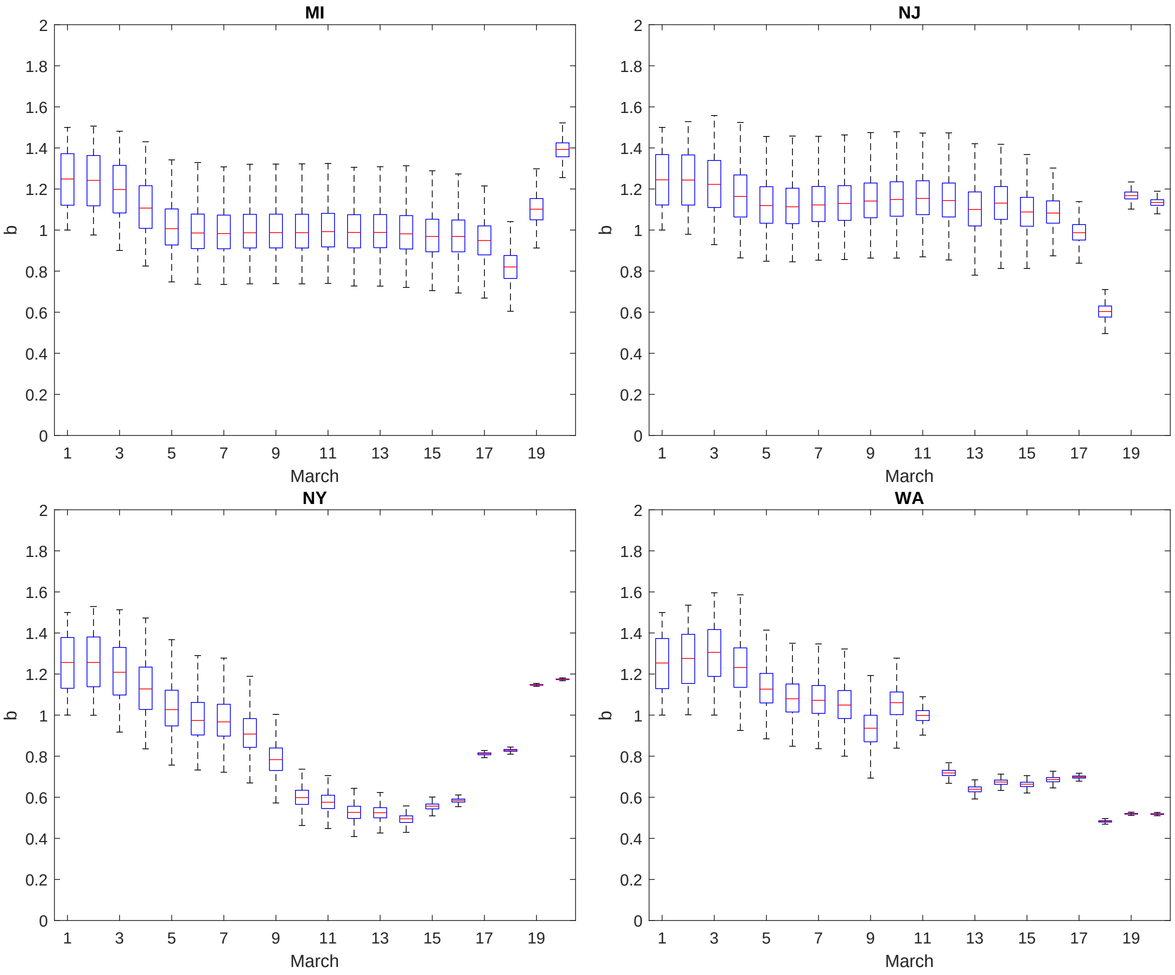

Figure S9: Inferred transmission rate b for different states. (continued)

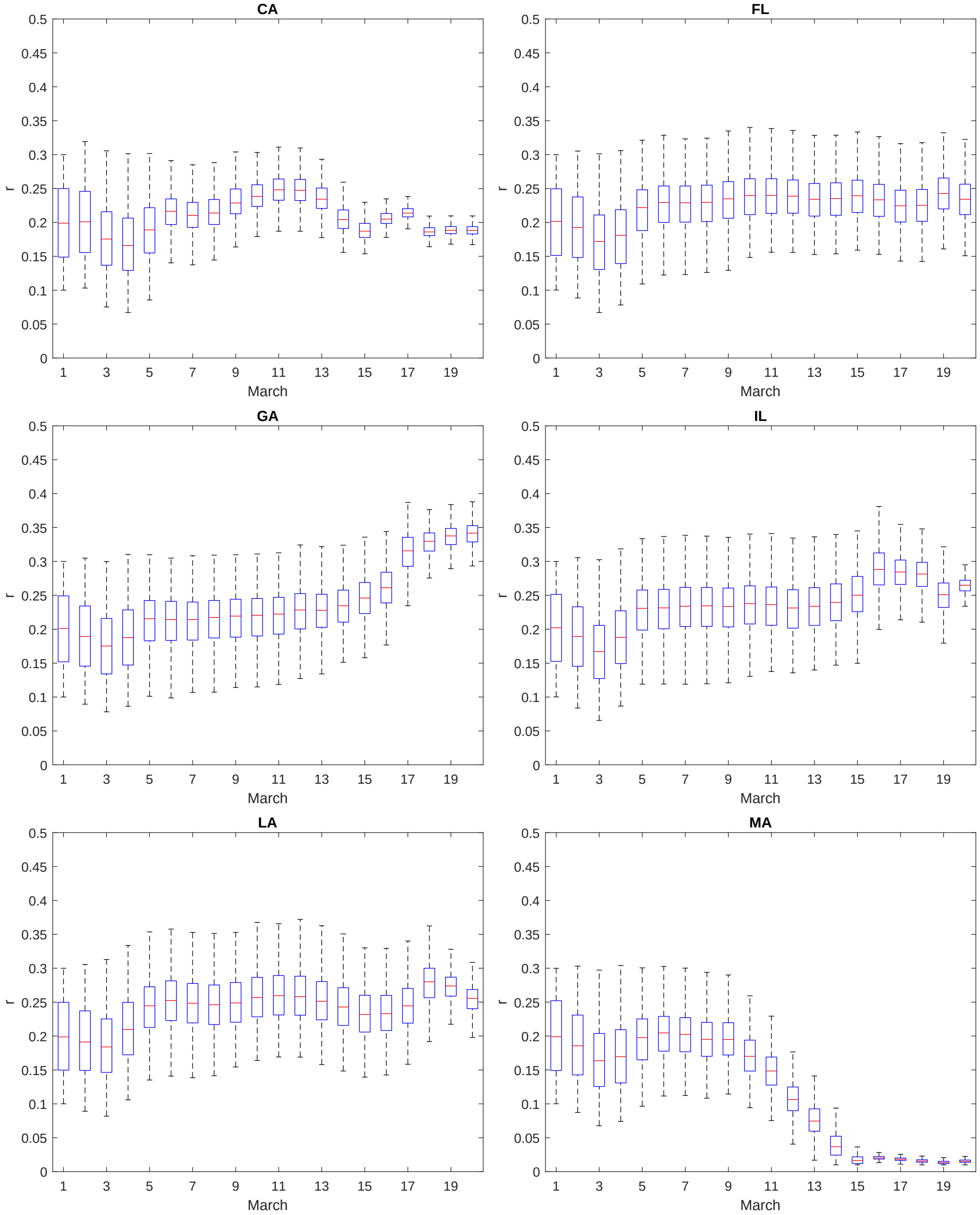

Figure S10: Inferred reporting rate r for different states. The box and whiskers show the median, interquartile range, and 95% credible intervals.

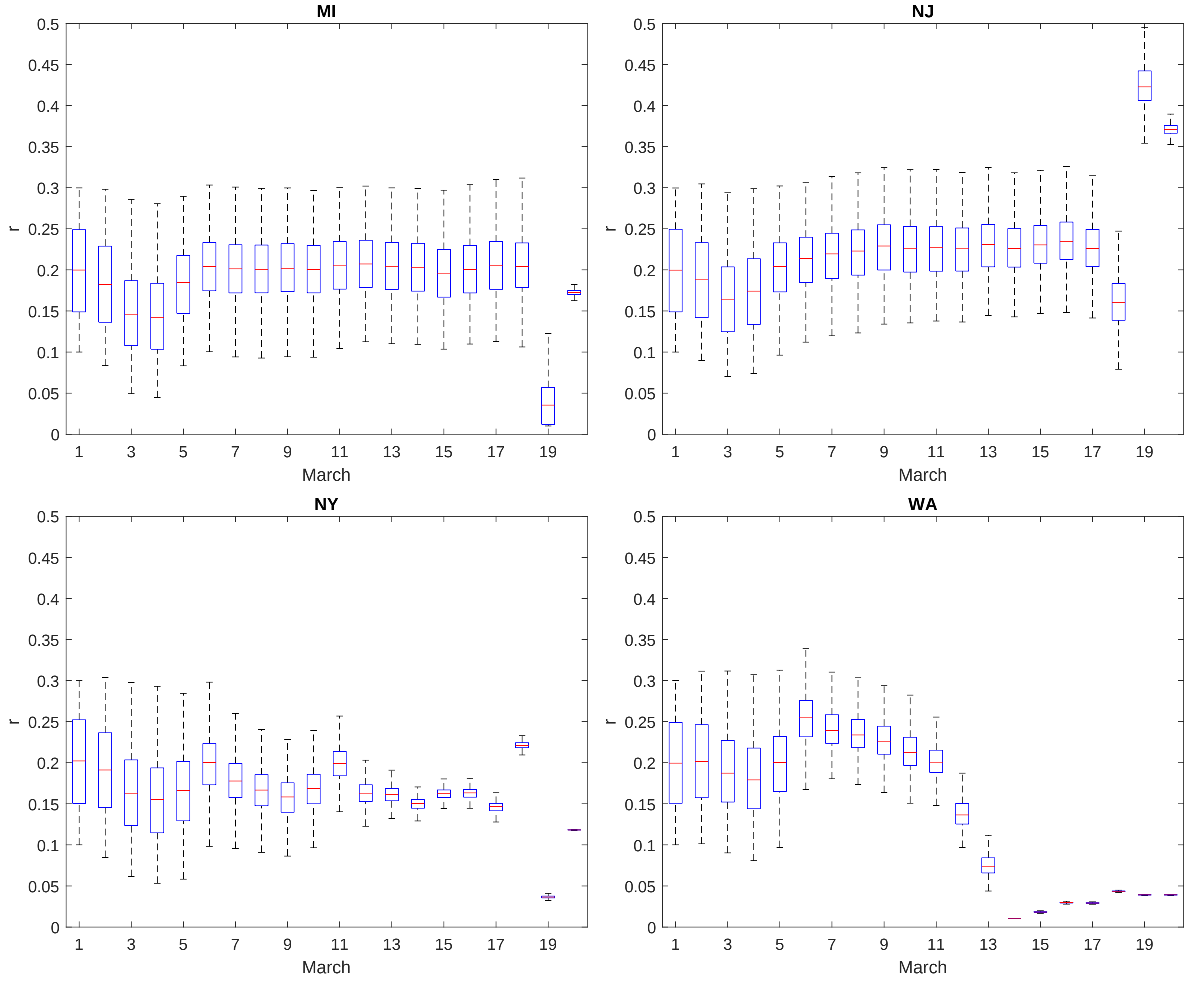

Figure S11: Inferred reporting rate r for different states. (continued)

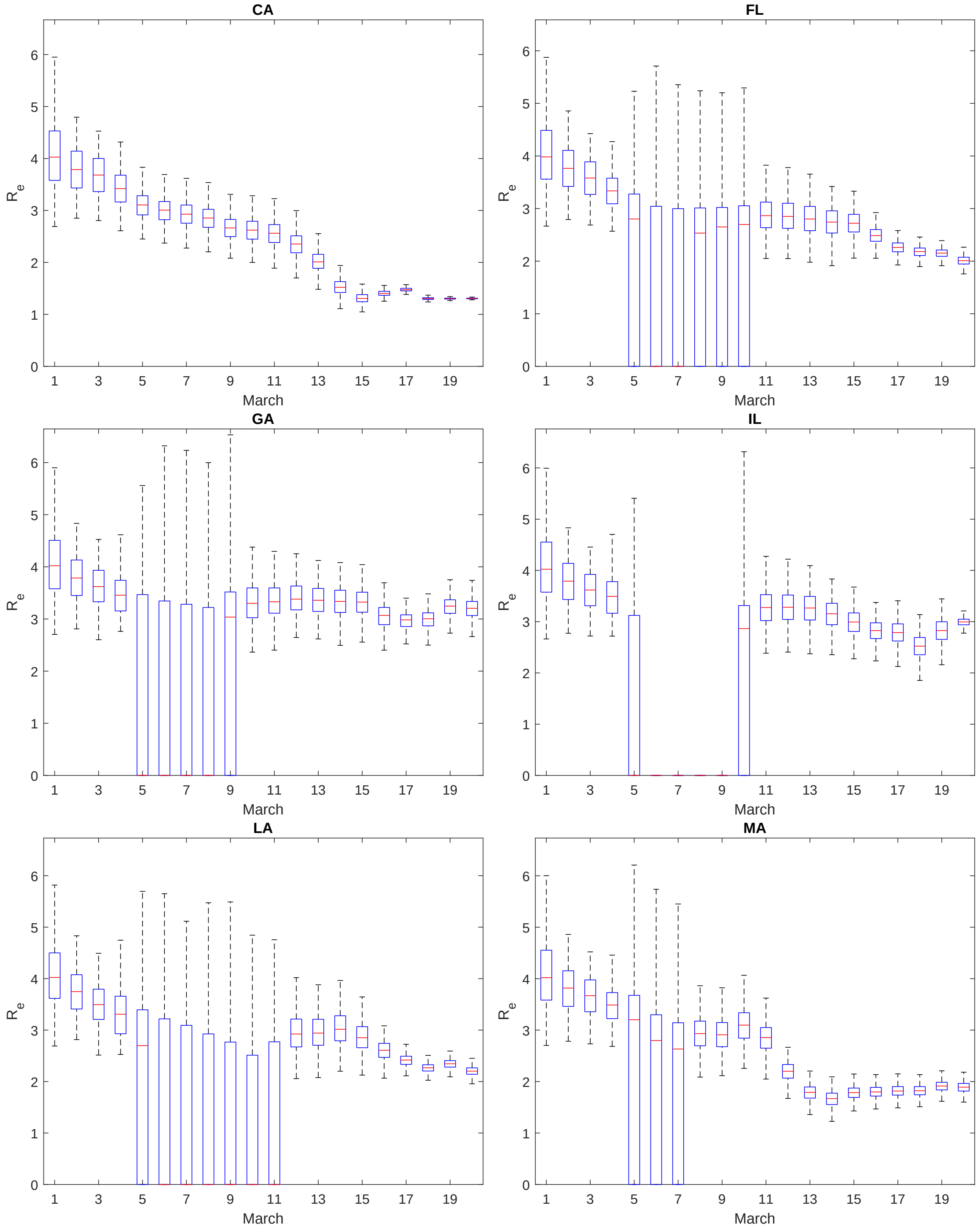

Figure S12: Inferred effective reproductive number R_e for different states. The box and whiskers show the median, interquartile range, and 95% credible intervals.

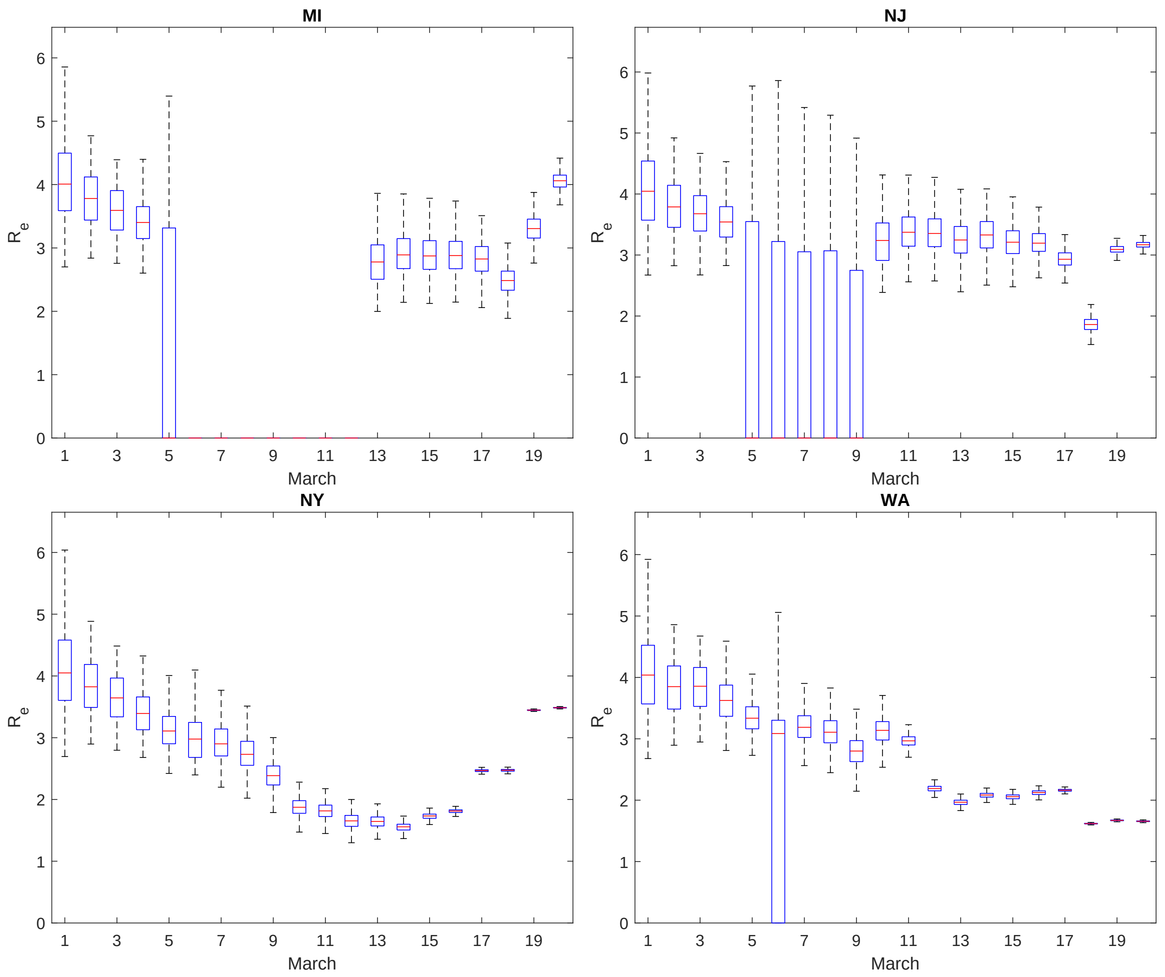

Figure S13: Inferred effective reproductive number R_e for different states. (continued)

4.2 Prediction

The first part of our prediction includes the case study for different transmission rate b and reporting rate r. In the following, we define the ratio between transmission rate in prediction step and data assimilation step (March 20) by α_b, and define the ratio between unreporting rate $1-r$ in prediction step and data assimilation step (March 20) by α_r, namely

$$b_i = \alpha_b b_{i,0}\,, \quad r_i = 1 - \alpha_r(1 - r_{i,0})$$

Fig S15 shows the effective reproductive number R_e on April 29 as a function of α_r and α_b for five states.

We further take a proactive approach by directly identify and quarantine the exposed population. The time in days used to discover and isolate them is, on average D_q. The model is changed to:

$$\begin{cases} \dfrac{\mathrm{d}S_i}{\mathrm{d}t} = -\dfrac{b_i S_i(U_i+\gamma E_i)}{P_i} + \sum\limits_{j\neq i} \alpha_t n_{ij}\dfrac{S_j}{P_j} - \sum\limits_{j\neq i} \alpha_t n_{ji}\dfrac{S_i}{P_i} \\ \dfrac{\mathrm{d}E_i}{\mathrm{d}t} = \dfrac{b_i S_i(U_i+\gamma E_i)}{P_i} - \dfrac{E_i}{D_q} + \sum\limits_{j\neq i} \alpha_t n_{ij}\dfrac{E_j}{P_j} - \sum\limits_{j\neq i} \alpha_t n_{ji}\dfrac{E_i}{P_i} \\ \dfrac{\mathrm{d}I_i}{\mathrm{d}t} = -c_I\dfrac{I_i}{D_c} - (1-c_I)\dfrac{I_i}{D_l} \\ \dfrac{\mathrm{d}U_i}{\mathrm{d}t} = -c_U\dfrac{U_i}{D_c} - (1-c_U)\dfrac{U_i}{D_l} + \sum\limits_{j\neq i} \alpha_t n_{ij}\dfrac{U_j}{P_j} - \sum\limits_{j\neq i} \alpha_t n_{ji}\dfrac{U_i}{P_i} \\ \dfrac{\mathrm{d}R_i}{\mathrm{d}t} = c_I\dfrac{I_i}{D_c} + (1-c_I)\dfrac{I_i}{D_l} + c_U\dfrac{U_i}{D_c} + (1-c_U)\dfrac{U_i}{D_l} \\ \dfrac{\mathrm{d}Q_i}{\mathrm{d}t} = \dfrac{E_i}{D_q} \end{cases} . \tag{4}$$

As shown in Fig. 3c, the timely self-quarantine and strict isolation right away or starting at most about 3.6 days (the median value) after exposed to the SARS-CoV-2 virus is found most effective in containing the COVID-19 outbreak for most contagious states in the US.

4.3 Limitation

For the scope of this paper, we do not consider the hospital capacity, and thus we assume the current or timely built facility can accommodate all reported infected cases. However, if one can have access to the hospital capacity data in each state, the capacity information can be fed to the model as well. Furthermore, we do not distinguish mild and severe symptoms in the model.

References

[1] CNN Report, Infected people without symptoms might be driving the spread of coronavirus more than we realized, available at https://www.cnn.com/2020/03/14/health/coronavirus-asymptomatic-spread/index.html.

[2] F. Brauer, *Compartmental models in epidemiology*, in Mathematical epidemiology, Springer, 2008, pp. 19–79.

[3] E. Dong, H. Du, and L. Gardner, *An interactive web-based dashboard to track COVID-19 in real time*, The Lancet Infectious Diseases.

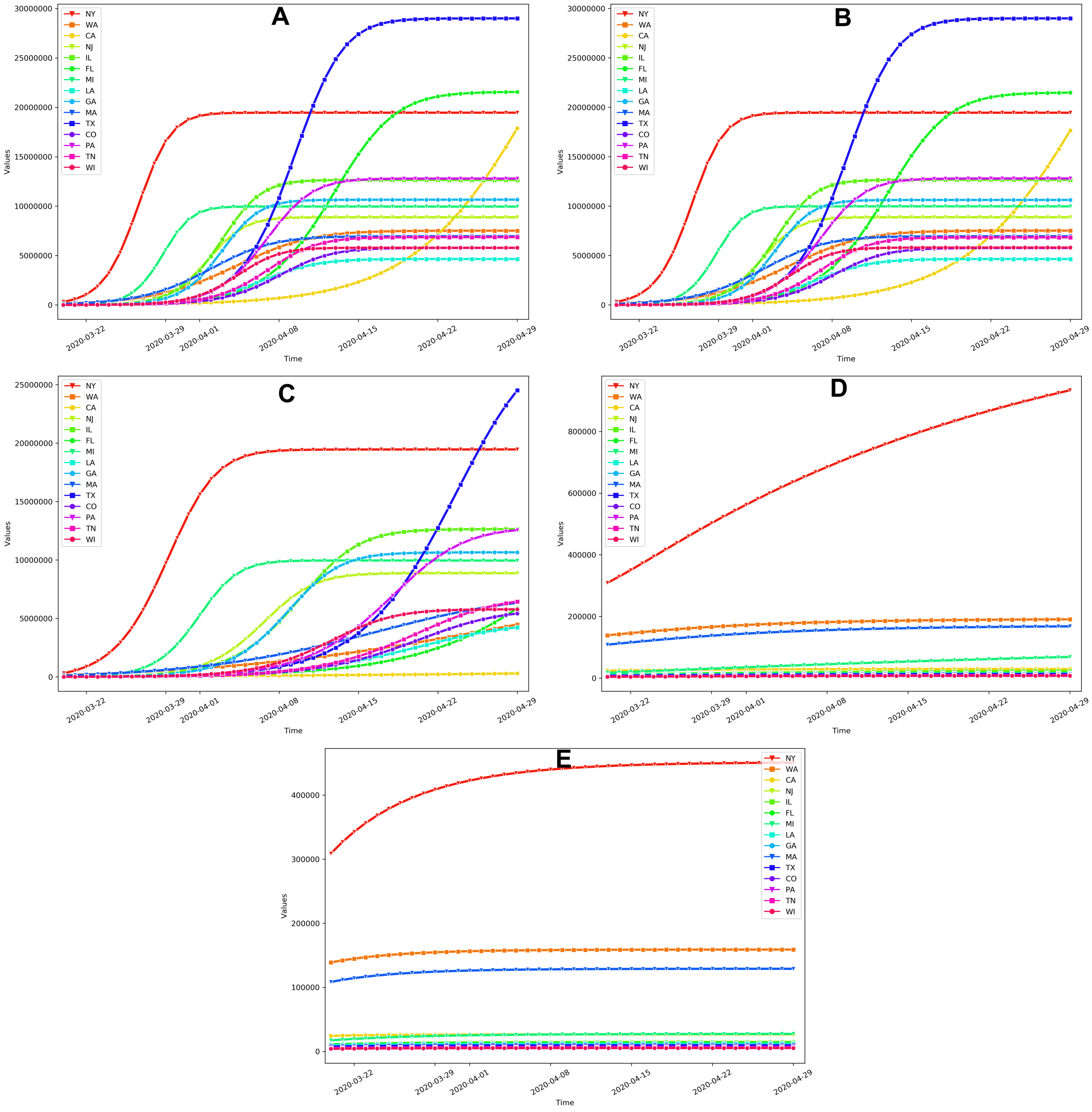

Figure S14: The prediction time series of the total infected population in the 15 most affected states under five scenarios: (A) $\alpha_r = 1$ and $\alpha_b = 1$, i.e., all parameters took the values of the initial configuration, obtained through data assimilation method using the numbers of confirmed cases during March 1 – March 20, 2020; (B) the travel flow was reduced to $\alpha_t = 0.05$, while other parameters values remained unchanged; (C) $\alpha_r = 0.1$ and $\alpha_b = 1$; (D) $\alpha_r = 1$ and $\alpha_b = 0.1$; (E) $\alpha_r = 0.1$, $\alpha_b = 0.1$. In the simulations, the transmission rate was set to be $b = \alpha_b b_0$ and the reporting rate was set to be $r = 1 - \alpha_r(1 - r_0)$. Where r_0 and b_0 were the reporting rate and the transmission rate on March 20, 2020, which are inferred from the data assimilation step.

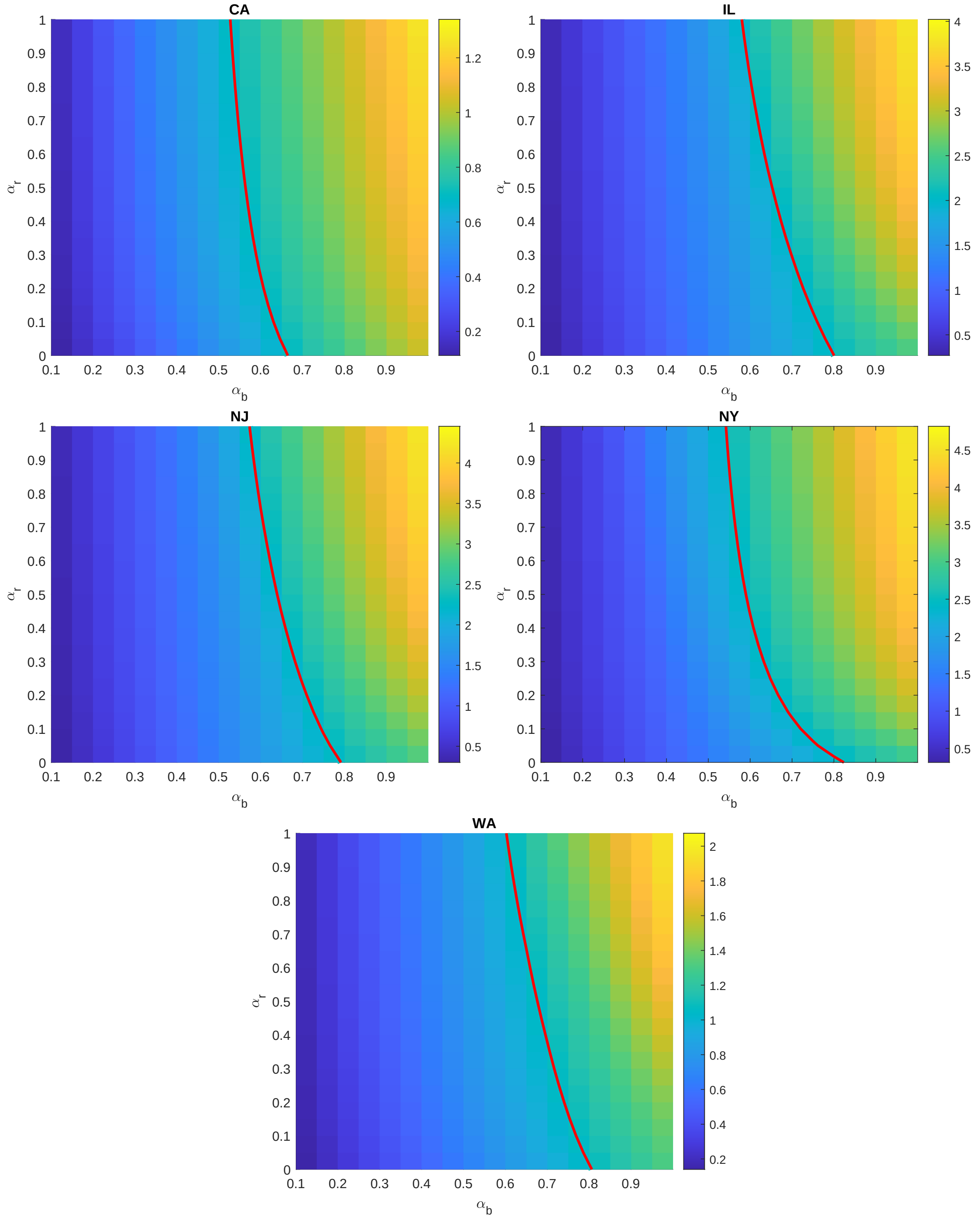

Figure S15: R_e on April 29 for different α_b and α_r. The red line is the level set $R_e = 1$. It can be seen that increasing the reported rate helps to diminish the reproductive number.

[4] G. Evensen, *The ensemble kalman filter for combined state and parameter estimation*, IEEE Control Systems Magazine, 29 (2009), pp. 83–104.

[5] W.-j. Guan, Z.-y. Ni, Y. Hu, W.-h. Liang, C.-q. Ou, J.-x. He, L. Liu, H. Shan, C.-l. Lei, D. S. Hui, et al., *Clinical characteristics of coronavirus disease 2019 in China*, New England Journal of Medicine, (2020).

[6] H. W. Hethcote, *The mathematics of infectious diseases*, SIAM review, 42 (2000), pp. 599–653.

[7] W. O. Kermack and A. G. McKendrick, *A contribution to the mathematical theory of epidemics*, Proceedings of the royal society of london. Series A, Containing papers of a mathematical and physical character, 115 (1927), pp. 700–721.

[8] S. Lai, N. W. Ruktanonchai, L. Zhou, O. Prosper, W. Luo, J. R. Floyd, A. Wesolowski, C. Zhang, X. Du, H. Yu, et al., *Effect of non-pharmaceutical interventions for containing the COVID-19 outbreak: an observational and modelling study*, medRxiv, (2020).

[9] N. H. Leung, D. K. Chu, E. Y. Shiu, K.-H. Chan, J. J. McDevitt, B. J. Hau, H.-L. Yen, Y. Li, D. KM, J. Ip, et al., *Respiratory virus shedding in exhaled breath and efficacy of face masks*, Nature Medicine, (2020).

[10] R. Li, S. Pei, B. Chen, Y. Song, T. Zhang, W. Yang, and J. Shaman, *Substantial undocumented infection facilitates the rapid dissemination of novel coronavirus (SARS-CoV2)*, Science, (2020).

[11] S. Reich and C. Cotter, *Probabilistic forecasting and Bayesian data assimilation*, Cambridge University Press, 2015.

[12] A. Stuart and K. Zygalakis, *Data assimilation: A mathematical introduction*, tech. rep., Oak Ridge National Lab.(ORNL), Oak Ridge, TN (United States), 2015.

[13] C. Wang, L. Liu, X. Hao, H. Guo, Q. Wang, J. Huang, N. He, H. Yu, X. Lin, A. Pan, et al., *Evolving epidemiology and impact of non-pharmaceutical interventions on the outbreak of coronavirus disease 2019 in Wuhan, China*, medRxiv, (2020).

[14] M. S. Warren and S. W. Skillman, *Mobility changes in response to COVID-19*, Descartes Labs, (2020).
